# On the Influence of Humidity on a Thermal Conductivity Sensor for the Detection of Hydrogen

**DOI:** 10.3390/s24092697

**Published:** 2024-04-24

**Authors:** Sophie Emperhoff, Matthias Eberl, Tim Dwertmann, Jürgen Wöllenstein

**Affiliations:** 1 Department of Microsystems Engineering (IMTEK), Albert-Ludwigs-Universität Freiburg, 79110 Freiburg im Breisgau, Germany; juergen.woellenstein@imtek.uni-freiburg.de; 2 Infineon Technologies AG, 81549 Neubiberg, Germanytim.dwertmann@gmail.com (T.D.); 3 Fraunhofer Institute for Physical Measurement Techniques (IPM), 79110 Freiburg, Germany

**Keywords:** thermal conductivity, hydrogen, water vapor, humidity, cross-sensitivity, MEMS

## Abstract

Thermal conductivity sensors face an omnipresent cross-influence through varying humidity levels in real-life applications. We present the results of investigations on the influence of humidity on a hydrogen thermal conductivity sensor and approaches for predicting the behavior of thermal conductivity towards humidity. A literature search and comparison of different mixing equations for binary gas mixtures were carried out. The theoretical results were compared with experimental results from three different thermal conductivity sensors with mixtures of water vapor in nitrogen. The mixing equations show a large discrepancy between each other. Some of the models predict a continuously decreasing thermal conductivity and some predict an increasing thermal conductivity for increasing levels of humidity. Our measurements indicate an increase in thermal conductivity followed by a decrease after reaching a peak value. It is shown that the measured behavior is reproducible with different sensors. Depending on the sensor, this corresponds to an error up to 2 vol.% in the measured hydrogen value. The measured behavior is consistent with only one of the three models. Compared to this model, our own sensor shows a maximum deviation of 1.4%. Mixing equations for gas mixtures must be chosen carefully, taking into consideration whether mixing partners include polar or non-polar molecules. Some simplified mixing equations cannot be used to calculate the thermal conductivity of water vapor in air or nitrogen.

## 1. Introduction

With increasing efforts being made to develop alternative drive systems, solutions such as electric and fuel cell vehicles are shifting into focus. Hydrogen as a fuel is seen as an important component of decarbonization in areas where electrification is not practical or possible [1]. Germany, for example, even provides a national hydrogen strategy to support achieving climate neutrality [2]. This development naturally leads to an increased interest in sensing solutions for hydrogen. The presence of hydrogen in concentrations between 4 and 77 vol.% can lead to explosive gas mixtures. Hydrogen leakage sensors can be used to avoid endangering passengers or bystanders in the event of leakages in a fuel cell vehicle. Battery monitoring can be another application for this type of sensor. In the event of a thermal runaway of a lithium-ion battery, various gases, including hydrogen, are released from the battery. In a study with 51 batteries, the concentration of hydrogen among the gases released was 22.27% on average [3]. That means hydrogen sensors can also be used to detect a thermal runaway event.

We can find many measurement principles for hydrogen sensors. Thermal conductivity is especially suited to hydrogen detection since the thermal conductivity of hydrogen is seven times higher than air at room temperature. Electrochemical or catalytic sensors may show higher sensitivities and a better selectivity towards hydrogen but also show drawbacks like a short lifetime and vulnerability to poisoning, respectively [4]. Applications like leakage detection of hydrogen in a vehicle require a certain lifetime and stability of the sensor which can be offered by a physical based measurement principle such as thermal conductivity. In safety relevant applications, the impact of cross-influences such as temperature, pressure and humidity also gain importance since they directly impact the accuracy of the sensor. In this study, we focus on the impact of humidity on thermal conductivity sensors. Humidity here corresponds to another gas in the gas mixture and thus contributes to the overall thermal conductivity. Thermal conductivity is a non-selective measurement principle as every material has its own thermal conductivity value. In the context of hydrogen measurements, this can lead to a distortion of the sensor signal if the proportion of humidity changes. Even if the hydrogen concentration remains constant, the sensor signal can either increase or falsely decrease due to an increased proportion of another gas, in this case moisture. This leads to problems, particularly in safety-relevant applications, as either false-positive or false-negative results can be obtained. We have investigated the influence of humidity theoretically and experimentally to determine the potential error of hydrogen sensor readings due to humidity.

This theoretical study provided numerous approaches for calculating the thermal conductivity of a gas mixture. With models for binary gas mixtures and models that have been introduced specifically for gas mixtures of water vapor and air, a theoretical comparison was carried out and then compared to experimental results. The available models use different input parameters or fitting parameters. We could identify a large discrepancy between those approaches. Most importantly, the expectation contradicted the measured behavior. The thermal conductivity of water vapor is lower than the thermal conductivity of dry air. Using a simple mixing equation based on the volumetric proportions of a gas mixture, this would lead to a decrease in the thermal conductivity of the overall mixture. It seems that many models cannot sufficiently describe the thermal conductivity of water vapor and air mixtures. This mixture of a polar and a non-polar gas leads to the more complex behavior of thermal conductivity due to the collision behavior of the molecules and their degrees of freedom.

In this study, we compared common mixing equations with measurements of our own thermal conductivity sensor for measuring hydrogen and compared them with other commercially available thermal conductivity sensors. We investigated the influence of humidity on different thermal conductivity sensors and evaluated whether common mixing equations can be used to describe and predict the behavior of those sensors.

Therefore, we conducted gas measurements between 25 and 85 °C with humidity variations between 0% and 95% relative humidity at atmospheric pressure. The results of all sensors were compared to the available mixing models.

## 2. Materials and Methods

### 2.1. Theoretical Background

Thermal conductivity is a material property that describes the ability of a material to conduct heat. Generally, the thermal conductivity of gases is very low compared to liquid or solid materials. Thermal conductivity values are often only available for pure gases, and data points for mixtures are limited. Since the target application requires measuring different concentrations of hydrogen in air, the focus is on the resulting thermal conductivity of a gas mixture. Measured values at a specific concentration, temperature, and pressure are hard to find. Therefore, methods with which the thermal conductivity of gas mixtures can be calculated are being sought.

In this study, two different gas mixtures were in focus, the target mixture of the sensor being an air–hydrogen mixture in order to investigate the cross-influence of a water vapor and air mixture. Both mixtures are considered binary gas mixtures in which air represents one component of the mixture (constant composition). In the literature, we can find different equations and approaches to estimating the thermal conductivity of binary gas mixtures. These equations are of varying complexity. We only concentrate on gas mixing equations for binary gas mixtures.

Udoetok presents a simple approach using the molar ratios of the gases and their corresponding thermal conductivity. The resulting thermal conductivity of the gas mixture is directly dependent on the molar ratios of the components in the gas mixture. Udoetok derives this from a model in which he considers thermal resistances and their parallel and serial connection [5].

(1) $$k_{mix}=0.5\times \frac{k_{A}k_{B}}{x_{A}k_{B}+x_{B}k_{A}}+0.5\times \left({x_{A}k}_{A}+x_{B}k_{B}\right)$$

where $k_{mix}$ is the resulting thermal conductivity of components *A* and *B* with their thermal conductivities $k_{A}$ and $k_{B}$, respectively. $x_{A}$ and $x_{B}$ are the molar fraction of each component in the mixture. This approach has already been investigated for binary gas mixtures of rare gases in a study by Mathur, Tondon and Saxena [6]. Zhukov and Pätz consider this approach for the calculation of a mixture of hydrogen and oxygen [7].

Mason and Saxena provide a general gas mixing equation which according to them is derived from kinetic theory by well-defined approximations [8]. Additional to the molar fractions and the thermal conductivities of the pure components, the molecular weights and viscosities at the corresponding temperature are needed for calculating a resulting thermal conductivity.

(2) $$k_{mix}=\frac{k_{A}}{1+\left(G_{AB}\frac{x_{B}}{x_{A}}\right)}+\frac{k_{B}}{1+\left(G_{BA}\frac{x_{A}}{x_{B}}\right)}$$

The parameters $G_{AB}$ and $G_{BA}$ are calculated using the viscosities $\mu_{A}$ and $\mu_{B}$ and molar masses $M_{A}$ and $M_{B}$ for the gases *A* and *B*, respectively.

(3) $$G_{AB}=\frac{\gamma }{2\sqrt{2}}\times {\left(1+\frac{M_{A}}{M_{B}}\right)}^{-0.5}\times {\left[1+{\left(\frac{\mu_{A}M_{B}}{\mu_{B}M_{A}}\right)}^{0.5}\times {\left(\frac{M_{A}}{M_{B}}\right)}^{0.25}\right]}^{2}$$

(4) $$G_{BA}=\frac{\gamma }{2\sqrt{2}}\times {\left(1+\frac{M_{B}}{M_{A}}\right)}^{-0.5}\times {\left[1+{\left(\frac{\mu_{B}M_{A}}{\mu_{A}M_{B}}\right)}^{0.5}\times {\left(\frac{M_{B}}{M_{A}}\right)}^{0.25}\right]}^{2}$$

This equation can be used for polyatomic gases in binary gas mixtures, but polar gases are excluded in the study of Mason and Saxena. The factor γ is an empirical factor and defined as 1.065 for non-polar gas mixtures [8].

Tsilingiris proposed a gas mixing equation for gas mixtures containing air and water vapor [9]. The base for this equation is the equation proposed by Mason and Saxena as described above. Tsilingiris adapted the empirical factor and conducted subsequent substitutions. He also added empirical equations to calculate the thermal conductivities and viscosities for water vapor and air for different temperatures.

(5) $$k_{mix}=\frac{(1-x_{v})\times k_{air}}{\left(1-x_{v}\right)+x_{v}\times \phi_{av}}+\frac{x_{v}\times k_{v}}{x_{v}+(1-x_{v})\times \phi_{va}}$$

With $x_{v}$ being the molar fraction of water vapor in air and the thermal conductivities $k_{air}$ and $k_{v}$ of air and water vapor, respectively. The paramterers $\phi_{av}$ and $\phi_{va}$ can be obtained as follows:

(6) $$\phi_{av}=\frac{\sqrt{2}}{4}\times {\left(1+\frac{M_{air}}{M_{v}}\right)}^{-0.5}\times {\left[1+{\left(\frac{\mu_{air}}{\mu_{v}}\right)}^{0.5}\times {\left(\frac{M_{v}}{M_{air}}\right)}^{0.25}\right]}^{2}$$

(7) $$\phi_{va}=\frac{\sqrt{2}}{4}\times {\left(1+\frac{M_{v}}{M_{air}}\right)}^{-0.5}\times {\left[1+{\left(\frac{\mu_{v}}{\mu_{air}}\right)}^{0.5}\times {\left(\frac{M_{air}}{M_{v}}\right)}^{0.25}\right]}^{2}$$

Melling et al. also proposed a model for calculating the thermal conductivity of a gas mixture containing air and water vapor [10]. The model includes a set of empirical equations to calculate the viscosities and thermal conductivities of air and water vapor at a temperature range from 100 °C and 200 °C. The gas mixing equation was taken from Mason and Saxena, but Melling et al. proposed a different empirical factor for this specific gas mixture containing a polar and a non-polar gas. Thermal conductivity is calculated using Equations (2)–(4), where the gases *A* and *B* represent air and water vapor, respectively. They state that a γ of 0.8 best fits the available measurement data.

Some of the presented models are based on the same original equation but use different values for the parameter γ. Melling et al. state that the most suitable value for this parameter is 0.8 for mixtures of polar and non-polar gases. Tsilingiris uses factor 1 for this polar and non-polar gas mixture of water vapor and air. Tondon and Saxena generally suggest a value of 0.85 for polar and non-polar gas mixtures [11]. Mason and Saxena use 1.065 in their standard equation for binary gas mixtures containing only non-polar gases.

Tondon and Saxena compared different approaches for calculating the thermal conductivity of polar and non-polar gas mixtures [11]. The conclusion of this study was that all four methods lead to an adequate calculation of thermal conductivity values with an error of around 2%. One method, which they call the approximate method, is the approach by Mason and Saxena from Equation (2), which is also used by Melling et al. Another approach among those investigated is the method by Lindsay and Bromley [12]. As they could not identify a clearly superior approach, our comparison does not include any further methods. The presented equations can be used for binary gas mixtures. Calculations of multicomponent gas mixtures have been performed by Muckenfuss and Curtiss and with a modification by Mason and Saxena [13,14].

**Figure 1:**
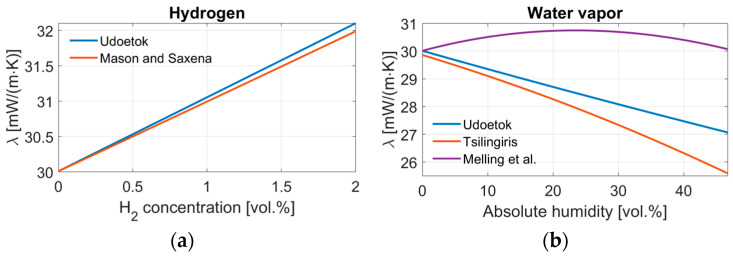
Comparison of the resulting thermal conductivity at 80 °C of gas mixtures containing (**a**) hydrogen in nitrogen as calculated by Equation (1) from Udoetok and Equations (2)–(4) from Mason and Saxena and (**b**) water vapor in air as calculated by Equation (1) from Udoetok, Equations (5)–(7) from Tsilingiris and Equations (2)–(4) using the empirical factor from Melling et al. [5,8,9,10].

Figure 1 presents a graphical comparison of the different mixing equations for a binary gas mixture containing hydrogen in nitrogen (a) and water vapor in air (b). Nitrogen is used as a substitute for air since air consists of 78 vol.% of nitrogen. Both mixing equations for hydrogen show a linear behavior for the selected range from 0 to 2 vol.% of hydrogen. The larger the hydrogen concentration, the larger the difference between both calculations. In the case of the water vapor and air mixture, it is important to notice that all three equations show a very different outcome. Since the thermal conductivity of water vapor is lower than that of air, the equation by Udoetok leads to a decrease in the thermal conductivity with an increasing molar fraction of water vapor. Tsilingiris also predicts a decrease in the thermal conductivity for higher concentrations of water vapor but at a different slope than Udoetok. A very different behavior is shown in the equation of Melling et al., where the thermal conductivity of the water vapor–air mixture first increases, reaches a maximum, and then decreases again.

### 2.2. Thermal Conductivity Sensors

The behavior of our MEMS-based thermal conductivity sensor towards humidity was investigated. The MEMS structure has already been introduced by Emperhoff et al. [15].

**Figure 2:**
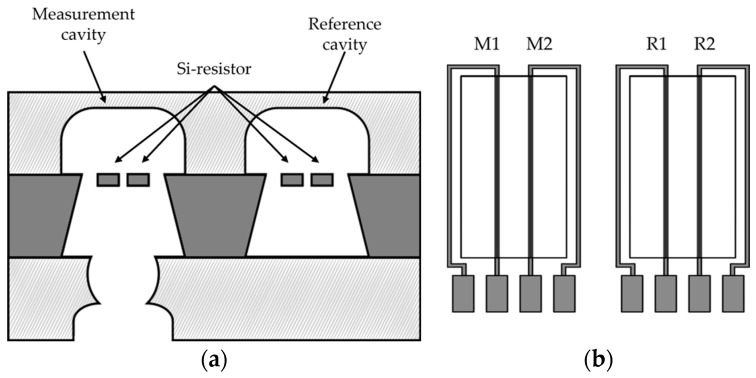
(**a**) Schematic cross-section of the MEMS structure used, as presented in [15]. (**b**) Schematic top view of the sensor as presented in [15]. The four resistors are connected as a Wheatstone bridge where two of them (M1, M2) are in a measurement cavity and the other two (R1, R2) are in a closed reference cavity.

The sensor structure will hereafter be referred to as the IFX sensor. It is based on a three-layer structure wherein a structured silicon wafer is sandwiched between two structured glass wafers. This stacking leads to two cavities with two silicon heaters each, which act as heater and resistor. One cavity is hermetically sealed through the two glass wafers, whereas the other cavity remains open with a gas inlet in the bottom wafer (Figure 2a). That means two resistors are used as a reference in a constant atmosphere and the other two resistors are exposed to gas changes. The resistors are connected as a Wheatstone bridge to allow a stable and low-noise measurement of resistance changes (Figure 2b). The resistors are both heaters and sensor elements. When a gas enters the open cavity, the thermal conductivity of the gas changes. This leads to a temperature change at the heater, as it influences how well the heat is dissipated. This causes a change in resistance, which can be measured via the Wheatstone bridge.

**Figure 3:**
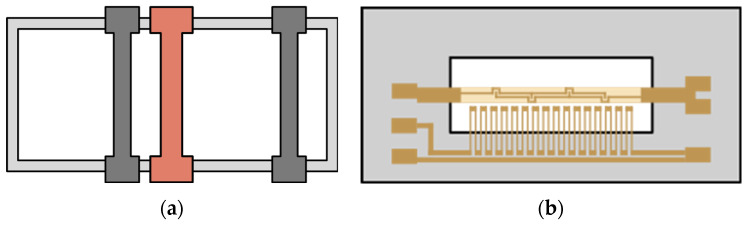
Schematic layout of reference thermal conductivity sensors with (**a**) the Sensirion STC31 on the left and (**b**) the Posifa Technologies PGS1000 on the right [16,17].

Additionally, two commercially available sensors with which to compare the IFX sensor were selected. These are the CO_2_ sensor STC31 by Sensirion (Sensirion AG, Staefa, Switzerland) and the H_2_ sensor PGS1000 by Posifa Technologies (Posifa Technologies, San Jose, CA, USA) [16,17]. All sensors are based on thermal conductivity as their measurement principle. Figure 3 shows a schematic overview of the two reference sensors. Figure 3a shows the structure of the Sensirion STC31 CO_2_ sensor. It is assumed that a heater structure in the middle is surrounded by two temperature-sensitive resistors at different distances from the heater. The PGS1000 hydrogen sensor consists of a heat source and a thermopile on a membrane which are above a gas cavity/heat sink (Figure 3b).

### 2.3. Gas Measuring Station

**Figure 4:**
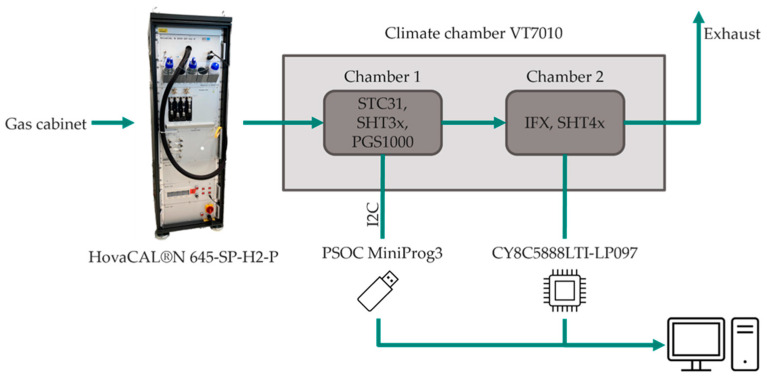
Schematic overview of the utilized measurement setup consisting of a gas-mixing unit (HovaCAL^®^) and a climate chamber containing two separate measurement chambers for the sensors to be tested.

All measurements were conducted using the gas measurement setup shown in Figure 4. The HovaCAL^®^ (IAS GmbH, Oberursel, Germany) is a calibration gas generator and can produce dry and defined humidified gas mixtures. The gas mixing unit is optimized for hydrogen gas mixtures and provides highly accurate hydrogen–nitrogen mixtures. The water dosage is administered via precise syringes and fed to the gas flow through an evaporator.

The preheated and humidified gas mixture is fed into the climate chamber via a heated line to avoid condensation. Two separate measurement chambers containing the devices under test are located within the climate chamber. Each chamber contains a temperature and humidity reference sensor to measure the ambient conditions in close proximity to the sensors. The Sensirion STC31 and the PGS1000 are placed in the first chamber. A humidity and temperature reference sensor are included in the STC31 module. Both sensors can be read out via an I2C bus. The communication is conducted with a PSOC MiniProg3 (Infineon Technologies AG, Neubiberg, Germany). The second chamber includes the IFX sensor and a SHT4x relative humidity sensor (Sensirion AG, Staefa, Switzerland). The IFX sensor is powered and read out via the Cypress microcontroller CY8C5888LTI-LP097 (Infineon Technologies AG, Neubiberg, Germany).

### 2.4. Measurement Methods

In this study, the behavior of thermal conductivity sensors towards humidity was investigated. To evaluate and compare the different thermal conductivity sensors, a calibration measurement with hydrogen in nitrogen was carried out first as all sensors deliver different sensor outputs. The STC31 is a CO_2_ sensor, and the output is given as a CO_2_ concentration in ppm. The PGS1000 is a hydrogen sensor which provides the hydrogen concentration as a percentage. The IFX thermal conductivity sensor provides a raw voltage signal.

The measurements were conducted in two steps. First, a calibration measurement was conducted using hydrogen in nitrogen with different concentrations. A humidity measurement with increasing water vapor concentrations in nitrogen followed the calibration measurement.

The measurements were carried out with a total flow rate of 1500 mL/min and repeated at different temperatures between 25 and 85 °C. The calibration measurement uses nitrogen as carrier gas and adds hydrogen to receive the concentrations 0%, 1%, and 2% of hydrogen. In the following measurements, the signal output of all sensors is converted into a hydrogen concentration using the measured sensitivity to hydrogen for each sensor at each temperature. Directly following the hydrogen measurement, the humidity measurement was conducted. The water vapor concentration was increased stepwise from 0 to 90% relative humidity.

The change in the original sensor output of each sensor is transformed into a hydrogen concentration on the one hand and a change in the thermal conductivity value on the other hand. Using the sensor sensitivity of the calibration measurement with hydrogen, all sensor signals are converted into a hydrogen equivalent. The expected thermal conductivity of a hydrogen–nitrogen mixture from 0 to 2 vol.% is calculated using the mixing equation by Mason and Saxena with a γ of 1.065, which, according to Zhukov and Pätz, is most suitable for hydrogen mixtures [7]. The change in the sensor signal of the hydrogen equivalent is then transformed into a corresponding change in thermal conductivity.

## 3. Results

### Humidity Response

Two out of three of the presented models predicted a decrease in thermal conductivity for increasing concentrations of water vapor. This means that there were large deviations between the results of those equations. We investigated how our thermal conductivity sensor behaved in actual measurement conditions. To verify the results, we not only tested the IFX sensor but also tested sensors from other producers, using hydrogen–nitrogen mixtures and water vapor–nitrogen mixtures. In the theory section, we saw that increasing the share of hydrogen in nitrogen leads to an increase in the thermal conductivity of the gas mixture. Within our measuring range, this behavior can be regarded as linear.

This means that according to two out of the three presented models, a signal change in the other direction is expected for humid gas mixtures. To simplify the presentation of the measurement results, the signals are displayed as hydrogen equivalents.

**Figure 5:**
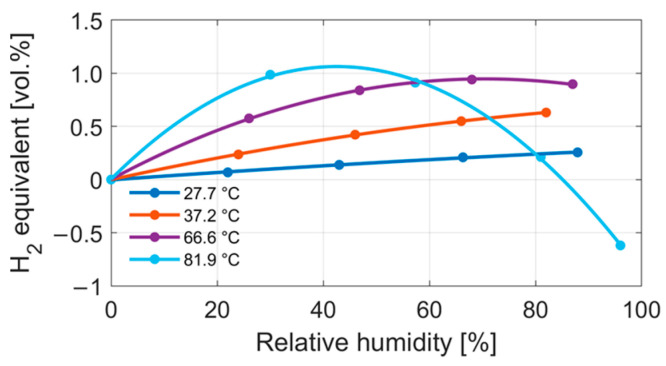
Humidity measurement of the IFX sensor at four different temperatures with increasing humidity levels between 0 and 90% relative humidity.

Initially, the IFX thermal conductivity sensor sample was measured alone to investigate the impact of humidity on the IFX sensor signal. Figure 5 shows the result of a characterization at four different temperatures, with increasing humidity levels between 0 and 90% relative humidity. The sensor output is displayed as a hydrogen equivalent at the respective temperature. The offset due to temperature changes is compensated. The first thing to note is that the sensor responded with a positive hydrogen equivalent for increasing levels of humidity. This indicates an increase in the thermal conductivity (contrary to what some mixing equations predicted). Furthermore, as temperature increased, so did the non-linearity of the sensor signal in response to the relative humidity. At some point, this even led to a turning point at which the signal decreased again and dropped below a 0% hydrogen equivalent. The higher the temperature, the larger the maximum hydrogen equivalent caused by the water vapor.

**Figure 6:**
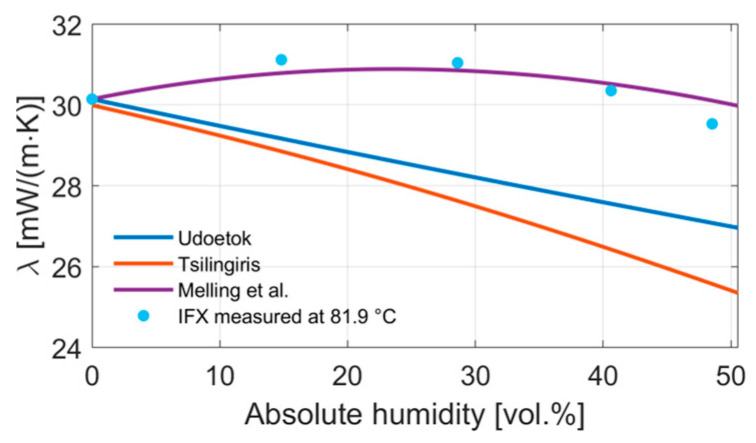
IFX sensor measurement compared to gas mixing equations from Udoetok, Tsilingiris and Melling et al. for the thermal conductivity of water vapor in air at 81.9 °C [5,9,10].

Figure 6 shows the measurement data at 81.9 °C compared to the introduced models. While comparing the models from the theory section with our measurement data, we can see that only the model by Melling et al. showed an increase in the thermal conductivity with increasing relative humidity [10]. The mixing equation of Udoetok and Tsilingiris resulted in a decrease in the thermal conductivity of water vapor and air mixtures, as was intuitively expected, since the thermal conductivity of water vapor is lower than that of air [5,9].

To ensure that this result was not just a misleading measurement or fault caused by the sensor system, the humidity measurement was repeated, and two external sensors based on thermal conductivity were added to the measurement setup as described in Figure 4. Those sensors are the STC31 CO_2_ sensor by Sensirion and the PGS1000 H_2_ sensor by Posifa Technologies. The measurements were performed as described in Section 2.

**Figure 7:**
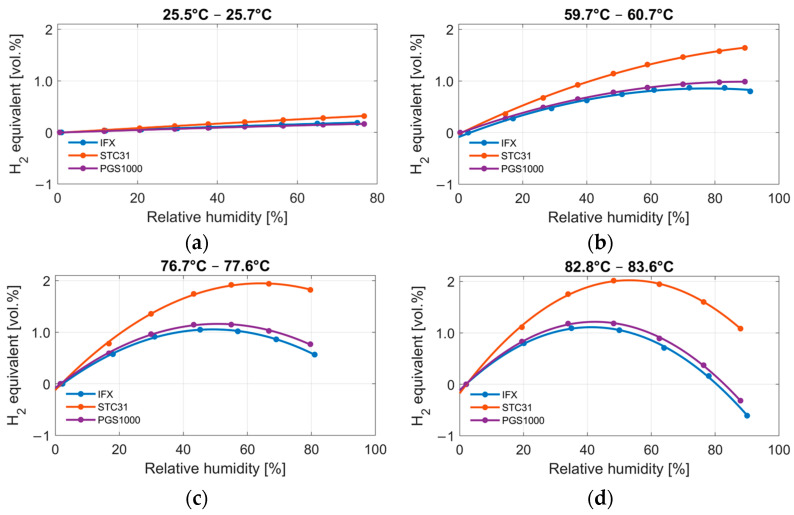
Measurement with three different thermal conductivity sensors (IFX, STC31 and PGS1000) at four different temperatures (**a**) 25.5–25.7 °C, (**b**) 59.7–60.7 °C, (**c**) 76.7–77.6 °C and (**d**) 82.8–83.6 °C, with varying humidity from 0 to 95% relative humidity.

Figure 7 shows the humidity measurements at four different temperatures. Mean values for every measurement point are displayed for each sensor. The results are given as hydrogen equivalents over the measured relative humidity. A polynomial curve of second order is fitted through the measurement points. The first thing to notice is that the humidity response parallels the hydrogen response for all three sensors. Second, the non-linearity increases at larger temperatures. Third, all three sensors show the same general behavior but with different magnitudes. The largest humidity response is shown by the STC31, which reaches up to 2 vol.% of hydrogen. The IFX and PGS1000 are quite comparable, especially at lower humidity levels, and show a maximum error between 1.1 and 1.2 vol.% of hydrogen.

**Figure 8:**
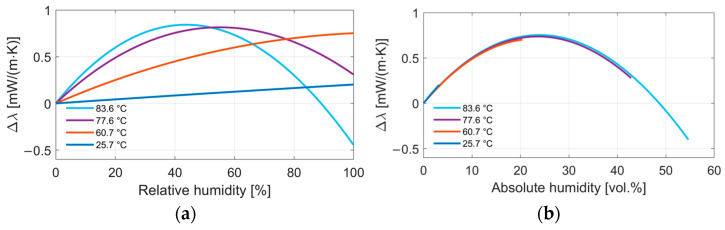
Result of the mixing equation by Melling et al. displaying the change in thermal conductivity of a water vapor–air mixture depending on (**a**) relative humidity and (**b**) absolute humidity in vol.% at four different temperatures between 25 and 85 °C [10].

Figure 8 displays the change in thermal conductivity as calculated with the equation from Melling et al. [10] Four different temperatures are displayed, depending on (a) the relative humidity between 0% and 100% (temperature offset compensated). The general behavior is quite comparable to what we see in the measurement results, with the two main characteristics of an increasing thermal conductivity and an increasing non-linearity at higher temperatures. Relative humidity results in a different absolute humidity at different temperatures; Figure 8b displays the thermal conductivity of a water vapor in air mixture depending on the absolute humidity in vol.%. The change in the thermal conductivity depending on the absolute humidity almost shows a parabola. That means that the change in thermal conductivity can be regarded as mostly dependent on the absolute humidity. The relative humidity is converted to an absolute humidity using following approach:

(8) $$AH=RH\times \frac{P_{s}(t)}{P_{b}}$$

The absolute humidity *AH* in vol.% is calculated using the relative humidity *RH* in %, the saturation vapor pressure $P_{s}$ in mbar at the reference temperature *t*, and the reference pressure $P_{b}$ in mbar. The saturation vapor pressure is calculated with the Goff–Gratch equation.

**Figure 9:**
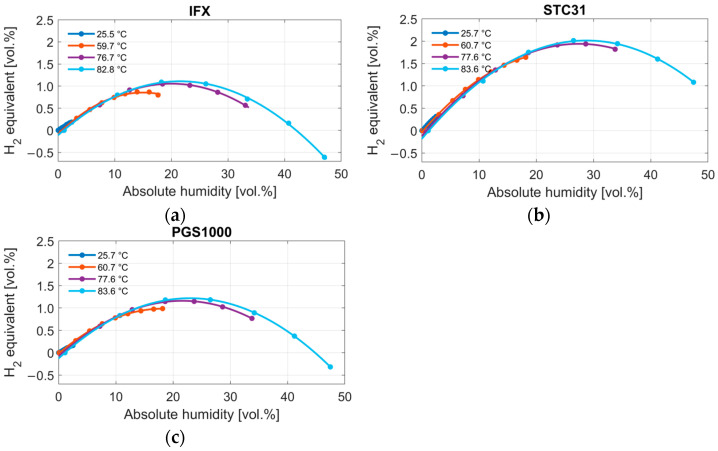
Result of the humidity measurement of the (**a**) IFX, (**b**) STC31, and (**c**) PGS1000 thermal conductivity sensors displayed as a hydrogen equivalent and depending on the absolute humidity in vol.%, as calculated using Equation (8).

Similar to the display of the model in Figure 8, Figure 9 shows the measurement results depending on the absolute humidity to evaluate whether the sensor output is in fact also mostly dependent on the absolute humidity share in air. We can see that the output seems to match well in low humidity ranges. However, at higher humidities, the results at the same absolute humidity but at different temperatures start to deviate. This applies to all three sensors, albeit to different degrees.

**Figure 10:**
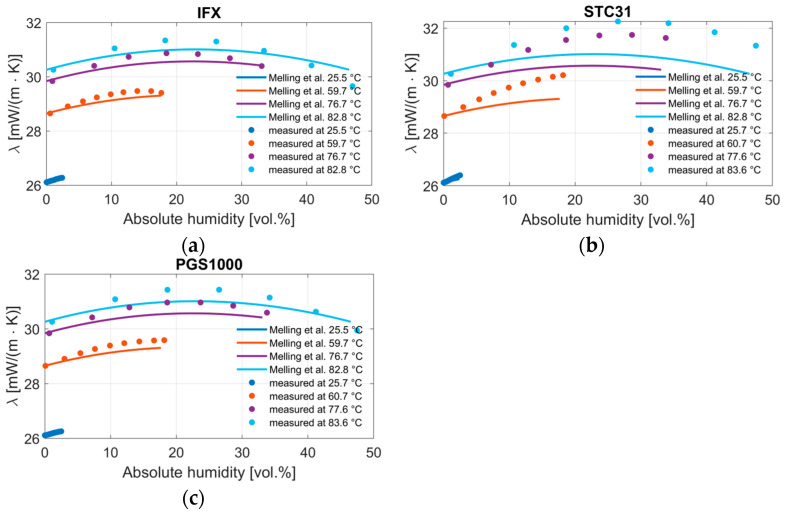
Thermal conductivity of water vapor and air mixtures at different temperatures as calculated with the equation of Melling et al. [10] compared to the measurement results of the (**a**) IFX, (**b**) STC31, and (**c**) PGS1000 thermal conductivity sensors. The results are displayed as a corresponding thermal conductivity value.

The same measurement data are transformed into a thermal conductivity value for a direct comparison to the model by Melling et al. [10]. The equation by Mason and Saxena is used to calculate a corresponding thermal conductivity change for a certain hydrogen concentration. This can be used to convert the hydrogen equivalent of the humidity measurements to an expected change in thermal conductivity. This delta is added to the thermal conductivity of 100% of the carrier gas at the respective temperature. Figure 10 shows the result of this display for all three sensors. The increase in the humidity level in the gas mixture seems to lead to an increase in thermal conductivity until the curve reaches its maximum and the thermal conductivity decreases again. Generally, the model shows the same behavior. A certain degree of uncertainty remains, particularly with the position of the maximum value and the magnitude, which is different for each sensor.

**Figure 11:**
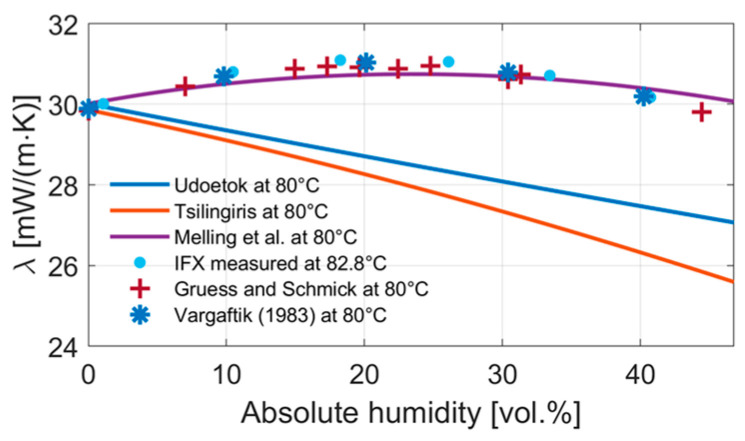
IFX measurements at 82.8 °C and different mixing equations for the thermal conductivity of water vapor in air at 80 °C compared to experimental data from Gruess and Schmick (1928) and Vargaftik (1983) taken from Tsilingiris [5,9,10,18,19].

To verify our measurement data, earlier measurement results by Gruess and Schmick and Vargaftik [18,19], which were taken from Tsilingiris [9], were compared to measured data from the IFX sensor as well as the presented mixing equations at a temperature of 80 °C (Figure 11). The IFX measurement was slightly above 80 °C with a temperature of 82.9 °C. The experimental data by Gruess and Schmick and Vargaftik are well in line with our own results.

## 4. Discussion

The three models presented showed different thermal conductivities for water vapor in air mixtures. Two of them are specifically for humid air mixtures. The third model is a simple mixing equation based on molar ratios. Intuitively, water vapor should decrease the thermal conductivity of the mixture since the thermal conductivity of pure water vapor is lower than the thermal conductivity of dry air. As a polar molecule with hydrogen bonds, water often behaves differently from other materials. During our measurements, we observed an increase in thermal conductivity with increasing levels of humidity, similar to what the mixing equation of Melling et al. indicated. At higher humidity levels, the thermal conductivity decreased again. Tsilingiris, on the other hand, predicted a decrease in thermal conductivity. The best alignment of experimental data with the models was achieved with the mixing equation from Melling et al. In general, experimental data for gas mixtures of water vapor and air are scarce for the intended temperature and pressure range. Experimental data were mostly cited from Gruess and Schmick and were taken from Tsilingiris to compare them with our own results. Tsilingiris also provides experimental data from Vargaftik. A deviation between experimental data and calculated values was acknowledged, but the model functioned sufficiently well. The smallest deviation between experimental and modeled data was found with the approach of Melling et al. [10]. This applies both to our own measurement results and to earlier data from Gruess and Schmick and Vargaftik which were taken from Tsilingiris [9,18,19]. When choosing a mixing equation for the thermal conductivity of gas mixtures, it is important to be aware of the boundary conditions. Mixtures of polar and non-polar gases cannot be predicted using simple equations such as the one from Udoetok [5]. We were able to show that the thermal conductivity of humid air first increases compared to dry air, although many publications still state that it decreases continuously.

Two commercially available sensors were investigated and compared to experimental results from our own sensor. All the investigated sensors are based on the measurement of thermal conductivity. Thermal conductivity sensors consist of a heat source and a temperature-sensing element which indicates changes in thermal conductivity through measuring temperature changes. Those two components can be separate, as in the STC31 and the PGS1000, or combined, as in the IFX sensor. If hydrogen or humidity are present in the vicinity of the sensing elements, the change in thermal conductivity causes a change in the temperature of the sensor element. In the case of the IFX sensor, this resulted in a change in resistance, which could be measured through the Wheatstone bridge. All three sensors qualitatively showed the same behavior towards humidity. The IFX and PGS1000 were also similar in the magnitude of the effect, which corresponded to 1.1 to 1.2 vol.% in hydrogen measurement values. The STC31 showed the largest effect towards humidity, with an equivalent of 2 vol.% in the hydrogen measurement value at maximum. The difference in the signal level can partly be explained by different heater temperatures. When the heater is activated, this creates a microclimate around the heater. This causes a temperature gradient between heater temperature and ambient temperature. Thermal conductivity is a temperature-dependent material property. The temperature gradient can be different for different gases. This can lead to an altered ratio of the thermal conductivities of hydrogen and water vapor. We assumed that the STC31 had the highest heater temperature compared to the other two sensors.

The behavior of all three sensors towards humidity fit best to the model proposed by Melling et al. [10]. The IFX, STC31, and PGS1000 showed a maximum error of 1.4%, 4.5%, 1.7%, respectively. There are several possible reasons for these deviations. The mixing models represent only thermal conductivity. In our measurements, we must also consider different types of temperature transport, which are always a combination of convection, conductivity, and heat radiation. Forced convection should be at a minimal level because of the sensors’ geometry and housing. Free convection, on the other hand, is present inside the measurement cavities. Furthermore, we must differentiate between thermal conductivity and thermal diffusivity. Thermal conductivity still contains the specific heat capacity and density of the gas. Mixing models cannot contain the whole complexity of those factors, especially since the extent of each factor varies with the sensor type and geometry.

## 5. Conclusions

In this paper, we investigated the impact of humidity on thermal conductivity sensors. This included a search of the literature for different mixing equations for calculating the thermal conductivity of binary gas mixtures and comparative measurements with different thermal conductivity sensors. Out study of the literature did not provide a clear result regarding the behavior of the thermal conductivity value for mixtures with varying humidity. Different approaches provide different solutions. Some models predict a continuous decrease and others an increase in thermal conductivity with increasing humidity, which is followed by a decrease at very high absolute humidities. However, the measurement results of three different sensors show a behavior comparable to the model of Melling et al. that indicates an increase followed by a decrease after thermal conductivity reaches a maximum. In our measurements, the sensor signals caused by humidity reached up to 2 vol.% in the hydrogen measurement value at maximum. The behavior of all three sensors was comparable, even if they displayed different degrees of change. Deviations between the experimental data of the three sensors and the model by Melling et al. [10] range between 1.4% and 4.5%. Earlier measurements by Gruess and Schmick or Vargaftik [18,19] are in line with these results. The behavior of the thermal conductivity depending on humidity is also shown in a study by Kimura, who even uses this behavior to measure the absolute humidity in air [20].

The results were compared with the identified mixing equations to evaluate whether these equations can be used to predict the behavior of thermal conductivity sensors and to show the actual behavior towards humidity for real-life applications. The thermal conductivity of the gas mixture containing water vapor and air or nitrogen first increased and then decreased again after it reached its maximum value. Simplified mixing models cannot be used to describe this behavior. Mixing models must be adjusted to fit the present gases, as in the approach by Melling et al., where the empirical factor of the equation by Mason and Saxena was adapted to a gas mixture containing water vapor and air [8,10].

More actual thermal conductivity measurements would be helpful for a more accurate adaption of the mixing equation to water vapor–air mixtures as the available experimental data are still limited in lower temperature and pressure ranges, as investigated in this study. Especially for measurements with the investigated sensors, we must also consider the microclimate of the heater structures, as the actual temperature around the heater does not correspond to the measured temperature and leads to a gradient surrounding the heater. Furthermore, measurements with the available sensors are not only influenced by thermal conductivity but could also include other types of temperature transport such as convection and heat radiation or sensor-specific characteristics and dynamic behaviors. The proposed gas mixing models cannot cover the whole complexity of those factors.

The results herein will help the implementation of thermal conductivity sensors in real-life applications. Thermal conductivity is mostly dependent on absolute humidity if it is temperature-compensated. It can be used as a simple way of compensating for humidity. We have shown that the observed behavior of our thermal conductivity sensor towards humidity is a general phenomenon that can be reproduced with other sensors and previous measurement data from other authors, even if some models still suggest a different behavior. Further investigations are planned to find adequate ways to improve models for sensor applications and to implement methods of compensating for humidity.

## Data Availability

Restrictions apply to the availability of these data. The datasets presented in this article are not readily available because of company restrictions. Requests to access the datasets should be directed to the corresponding author.
